# Atezolizumab and Bevacizumab Attenuate Cisplatin Resistant Ovarian Cancer Cells Progression Synergistically via Suppressing Epithelial-Mesenchymal Transition

**DOI:** 10.3389/fimmu.2019.00867

**Published:** 2019-04-26

**Authors:** Lei Zhang, Ying Chen, Fangxuan Li, Lewen Bao, Wenxin Liu

**Affiliations:** ^1^Department of Gynecologic Oncology, Tianjin Medical University Cancer Institute and Hospital, Tianjin, China; ^2^Key Laboratory of Cancer Prevention and Therapy, Tianjin, China; ^3^National Clinical Research Centre of Cancer, Tianjin, China; ^4^Department of Cancer Prevention, Tianjin Medical University Cancer Institute and Hospital, Tianjin, China

**Keywords:** PD-L1, Atezolizumab, angiogenesis, Bevacizumab, ovarian cancer

## Abstract

The AURELIA trial demonstrated that adding Bevacizumab to chemotherapy significantly improved progression-free survival (PFS) for platinum resistant recurrent ovarian cancer. Recently, immunotherapy also presented potential anti-tumor effects in several malignant solid tumors. This study aimed to investigate whether combining anti-PD-L1 Atezolizumab with BEV may have a synergistic effect and enhance the efficacy of both treatments in cisplatin resistant epithelial ovarian cancer (CREOC). We retrospectively analyzed 124 epithelial ovarian cancer (EOC) patients from Gynecologic Oncology Department of Tianjin Cancer Hospital between January 2013 and June 2018, who all were diagnosed with cisplatin resistance due to progressing <6 months after completing platinum-based therapy. Based on responding to at least 2 cycles of Bevacizumab-containing chemotherapy (BC), these Patients were divided into BC response group and BC non-response group. Immunohistochemistry was used to detect that PD-L1 expression and tumor angiogenesis-related proteins (VEGF and Semaphorin4D) in tissues from 124 patients with CREOC. The positive expressions of PD-L1, VEGF, and Semaphorin4D (SEMA4D) were found in 58.73, 50.79, and 71.43% of the 63 cases CREOC tissues with BC response, respectively, which were significantly higher than that in the 61 cases BC non-response group (*P* < 0.05). PD-L1 expression correlated with SEMA4D and VEGF positively (*r* = 0.344 and 0.363, *P* < 0.001). Over-expressions of PD-L1, VEGF and SEMA4D are associated with more malignant clinicopathologic characteristics of CREOC Patients. In survival analysis, patients' response to BC was the independent factor for evaluation of PFS and overall survival (OS). Cell functional assays showed that Atezolizumab in combination with Bevacizumab inhibited the proliferation, migration, and invasion of cisplatin resistant ovarian cancer cell line A2780cis *in vitro* synergistically, which maybe associate with Bevacizumab suppressing the epithelial-mesenchymal transition (EMT) and PD-L1 expression by targeting STAT3. Furthermore, Bevacizumab and Atezolizumab induced synergistic anti-tumor effect *in vivo*. These findings suggest a novel therapeutic strategy for cisplatin resistant recurrent EOC and its mechanism warrants further study.

## Introduction

Advanced epithelial ovarian cancer (EOC) is still characterized by a poor prognosis despite the dramatic improvements have achieved under the therapeutic strategies of paclitaxel in combination with carboplatin followed by debulking (1). Poor survival is thought to be attributed to various factors including the non-specific symptoms of the initial disease, the advanced disease stage at diagnosis and acquisition of chemo-resistance following treatment (2). There is therefore a need for novel, more targeted and personalized therapies for cisplatin resistant EOC (CREOC) patients.

Recently, adding anti-angiogenesis treatments expose potential encouraging results in different tumor types. Bevacizumab, a monoclonal antibody binding vascular endothelial growth factor (VEGF), has been recently incorporated in the treatment of ovarian cancer patients after multiple clinical trials have proven its clinical benefit, even for cisplatin resistant recurrent ovarian cancer (3, 4).

Immunotherapy is another exciting new avenue and its use is gaining huge momentum in oncology with immune checkpoint inhibitors showing great promise in several cancers (5). Programmed cell death 1 ligand 1(PD-L1), a coinhibitory factor that is expressed on many types of cancer cells, by binding to its receptor, programmed death 1 (PD-1) on lymphocytes, PD-L1 transmits a signal that inhibits lymphocyte activation to promote cancer progression. The efficacy of Atezolizumab, an anti-PD-1 antibody, in multiple cancer types had already been reported (6).

The semaphorin (SEMA) superfamily is a class of proteins sharing a common Sema region, which also has been proved to be involved in the process of tumor angiogenesis (7). Semaphorin4D (SEMA4D), one of the members of SEMA IV subfamily, promotes angiogenesis in several cancers (8). Previous studies had identified SEMA4D and VEGF had a positive correlation in EOC cancer tissues and knockdown of VEGF could suppress SEMA4D expression, which indicate poor prognosis for EOC patients (9, 10).

At present, it reached a consensus that the combination of different therapeutic strategies was becoming the way forward in the field of cancer treatment (11). In this study, we explored the expressions of angiogenesis molecules (VEGF and SEMA4D) and PD-L1 as well as their relationships and prognostic roles in CREOC. Bevacizumab and Atezolizumab are hypothesized to have a synergistic inhibitory effect on the malignant behaviors of CREOC cell line A2780cis *in vitro* and tumor growth *in vivo*. The underlying mechanisms of resistant cancer cell progression mediated by BEV and Atezolizumab were investigated.

## Materials and Methods

### Patients and Specimen

With approval from the Institutional Review Board at Tianjin Cancer Hospital, we retrospectively screened consecutive patients who had been diagnosed pathologically as EOC between January 2013 and June 2018. The inclusion criteria for this study is followed: ➀ All patients received primary surgery; ➁ All primary tumor tissue samples were obtained at the time of primary surgery. ➂ All patients received platinum based first-line chemotherapy after primary surgery; ➃ All patients were evaluated recurrent ovarian cancer as resistant to platinum; ➄ All patients received single regimen chemotherapy plus Bevacizumab in the second-line treatment after diagnosed recurrence; ➅ Bevacizumab treatment was carried on at least 2 cycles.

**Figure 1:**
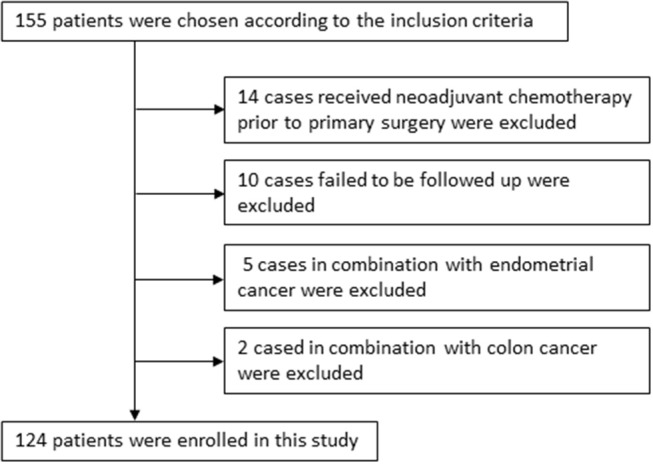
124 epithelial ovarian cancer patients with cisplatin resistant recurrence enrolled in this study were described graphically.

One hundred and fifty five cases were chosen according to the inclusion criteria. Fourteen samples from patients who received neoadjuvant chemotherapy prior to primary surgery and 10 cases failed to be followed up were excluded. Five cases with endometrial cancer were excluded and 2 with colon cancer were excluded. Finally, 124 cases were enrolled in this study. The detailed clinical data were collected from retrospective reviews of the patients' medical charts, which were summarized in Table 1 and described graphically in Figure 1. Overall survival (OS) time was calculated from the date of surgery to the date of death or last follow-up (at which point the patient was censored). Progression-free survival (PFS) was defined as the time elapsed from the date of completing second-line therapy to the appearance of disease recurrence or progression (failure) or the last follow-up for women who were alive with no evidence of disease recurrence or progression (censored). All patients were followed until death or the end of the follow-up period (August 31, 2018). Patients were staged using the International Federation of Gynecology and Obstetrics (FIGO) 2009 staging system and were further divided into early stage (I–II) and advanced stage (III–IV) for the purpose of statistical analysis. Optimal debulking surgery was defined as residual disease <1 cm. All patients provided written informed consent for the use of their tissues.

**Table 1:** Characteristics of ovarian cancer patients.

**Characteristic**	**Cases N (%)**
Age (mean ± SD, year)	Median: 52; Range: 26–78
≤52	62 (50.0)
>52	62 (50.0)
BMI (kg/m^2^)	23.79 ± 4.02
Menopause	
Yes	76 (61.3)
No	48 (38.7)
Pathologic type	
Serous	82 (66.1)
Mucous and others	42 (33.9)
Figo stage	
I–II	51 (41.1)
III–IV	73 (58.9)
Histologic grade	
G1-2	47 (37.9)
G3 and undifferentiated	77 (62.1)
Residual disease	
<1 cm	93 (75.0)
≥1 cm	31 (25.0)
Ascites volume (mL)	Median: 2,000; Range: 200–8,000
<2,000	103 (83.1)
≥2,000	21 (16.9)
Serum CA125 (U/mL)	Median: 573.35; Range: 14.10–67.62
<573.35	74 (59.7)
≥573.35	50 (40.3)
LN metastasis	
No	76 (61.3)
Yes	48 (38.7)
Response to BC after 2 cycles	
Response (CR, PR, and SD)	63 (50.8)
Non-response (PD)	61 (49.2)

*BC, bevacizumab plus single regimen chemotherapy; CR, Complete Response; PR, Partial Response; PD, Progressive Disease; SD, Stable Disease*.

### Cells and Reagents

Ovarian cancer cell line A2780 was purchased from the Chinese Academy of Sciences (Shanghai, China). The cisplatin-resistant ovarian cancer cell line A2780cis was purchased from ECACC (European Collection of Cell Cultures, Salisbury, UK). A2780 cells were cultured in RPMI 1640 medium (Invitrogen, Carlsbad, CA, USA) supplemented with 10% fetal bovine serum (FBS). A2780cis cells were cultured in RPMI 1640 + 2 mM Glutamine + 1 μM cisplatinum + 10% FBS. Cells were cultured in humidified air at 37°C in an atmosphere of 5% CO2. Atezolizumab (Tecentriq®) was purchased from Roche Inc. BEV was provided by the pharmacy of Tianjin Cancer Hospital (Roche, Switzerland).

### Immunohistochemistry

Sections of 4-mm thick serially cut from formalin-fixed and paraffin-embedded tissue blocks were deparaffinized, rehydrated and autoclave-treated at 121°C for 10 min in 0.1 M citrate buffer (pH 6.0) to induce antigen retrieval. Endogenous peroxides in the section were blocked by incubation in 3% hydrogen peroxide for 5 min. Then, after being blocked with 0.5% goat serum for 60 min, the sections were incubated at 4°C overnight with PD-L1 (Cell Signaling Technology, Inc., Leiden, Netherlands) and VEGF primary antibodies (Santa Cruz Biotechnology Inc., Santa Cruz, CA, USA) or with SEMA4D (BD Biosciences, San Jose, CA, USA). The DAKO REAL EnVision Detection kit (DAKO) was subsequently applied for 30 min. Finally, sections were incubated in 3,3′-diaminobenzidine for 5 min, followed by Mayer's hematoxylin counterstaining and mounting. Negative controls were obtained by replacing the primary antibody with isotype-matched monoclonal antibody. The percentage of positive cells was rated on the following point scale: no points (negative), ≤10% positive cells, regardless of staining intensity; 2 points, 11–50% positive cells; 3 points, 51–80% positive cells; and 4 points, ≥81% positive cells. The staining intensity was rated as follows: 1point, weak intensity; 2 points, moderate intensity; and 3 points, strong intensity. Points for the percentage of positive cells and staining intensity were added, and specimens were attributed to two groups according to their overall score. Finally, specimens of ≤3 points were rated as negative, or as positive. Two independent investigators blinded to clinical data performed the analysis.

### Apoptosis Analysis Using Flow Cytometry

Apoptotic cells were assessed according to the protocol of the Annexin V-FITC Apoptosis Detection Kit as described previously. Briefly, the cells were seeded in 6-well plates and were treated with different concentration of cisplatin (20, 40, and 60μM) for 24 h. After harvesting, the cells were resuspended in 500 μl of binding buffer supplemented with 5 μl of AnnexinV-FITC a nd 5 μ l o f propidium iodide (PI). Finally, the cells were analyzed using flow cytometry (Becton Dickinson, USA).

### Cell Proliferation Assay

A2780cis cells (1 × 10^4^ per well) with different four groups [➀ control; ➁ Bevacizumab 5.0 μg/mL; ➂ Atezolizumab 10.0 μg/mL; ➃ Bevacizumab 5.0 μg/mL; and Atezolizumab 10.0 μg/mL] were cultured in 96-well plates. Proliferation was assessed using the Cell Counting Kit-8 (Dojindo, Kumamoto, Japan) at 1, 2, 3, 4, and 5 days according to the manufacturer's instructions. Absorbance was determined at 450 nm using an Elx800 reader (Bio-Tek Instruments Inc., Winooski, VT, USA). Inhibition rate of cell proliferation was calculated by formula (1−OD^experimentgroup^/OD^control^)^*^100%.

### Cell Invasion Assay

The invasion of 4 different group cells was assessed by Transwell chambers precoated with Matrigel (BD, Franklin Lakes, NJ, USA). After incubation for 48 h, cells on the upper surface of the Transwell inserts were removed and cells on the lower surface were fixed and then stained with 0.5% crystal violet solution. Cells were counted in five random fields in each well.

### Wound Healing Assay

Cells were cultured in RPMI 1640 + 2 mM Glutamine + 1 μM cisplatin + 10% Fetal Bovine Serum (FBS) in 35 mm Petri dishes. Once cells reach 90% confluence media was aspirated and cells were washed twice with PBS. Using a 200 μL pipette tip three separate wounds were created across the monolayer culture perpendicular to the bottom line. The A2780cis cells were maintained to culture by using of the starvation media of RPMI 1640 + 2mM Glutamine + 1 μM cisplatin + 1% FBS with different treatment [➀ control; ➁ Bevacizumab 5.0 μg/mL; ➂ Atezolizumab 10.0 μg/mL; ➃ Bevacizumab 5.0 μg/mL and Atezolizumab 10.0 μg/mL], respectively. After 48 h culture, media were discarded and cells were washed twice with PBS. The distances of wound were determined at different time intervals (0, 24, and 48 h).

### Western Blot Analysis

Proteins from cells were separated by sodium dodecyl sulfatepolyacrylamide gel electrophoresis (SDS-PAGE) and then immunodetection was performed with standard techniques. Antibodies to E-cadherin, vimentin, β-catenin, Snail, Slug, and β-actin were purchased from Santa Cruz Biotechnology, Inc. (Santa Cruz, CA, USA). Antibodies for phosphorylated-STAT3 (p-STAT3) and STAT3 were from Cell Signaling Technology (Beverly, MA, USA). Signals were visualized with SuperSignal® West Pico Chemoluminescent Substrate (Pierce, Rockford, IL, USA) by exposure to films. The results were evaluated by Image J software, which were showed with bar graphs.

### *In vivo* Studies

Female BALB/C nude mice were purchased from Charles River Japan (Tokyo, Japan). Animal experiments were approved by tianjin medical university cancer hospital and institute animal research committee and animals were maintained under specific pathogen-free conditions. To evaluate the effect of Bevacizumab and Atezolizumab on tumor growth, A2780cis cells (5 × 10^6^) were injected subcutaneously into the right shoulders of syngeneic mice. One week later after injection, the graft tumor reached 9~10 mm^2^. And then, the mice were divided into 4 groups and there were six mice in each group. The treatment for each group was started and as follows: ➀ IgG as control; ➁ Bevacizumab (5 mg/kg) every 48 h; ➂ Atezolizumab (10 mg/kg) every 48 h; ➃ Bevacizumab (5 mg/kg) + Atezolizumab (10 mg/kg) every 48 h. The treatment was performed every other day and Mice were killed after treating for 3 weeks. Tumor size was calculated every other day and the volume of the tumor was estimated using the following formula: Estimated tumor volume = length × width (mm^2^).

### Statistical Analysis

The spearman rank correlation and Mantel-Haenszel test were used to assess the degree of correlation among variables. The survival rate was determined by the Kaplan-Meier method, and the log rank test was used to determine significance. Factors that were deemed of potential importance by univariate analysis were included in the multivariate analysis. A result was considered significant when the *p* value was < 0.05. All statistical analysis was performed with SPSS version 17.0 (SPSS Inc., Chicago, IL, USA).

## Results

### Higher Expressions of PD-L1, SEMA4D, and VEGF in Ovarian Cancer With BC Response Than Those With BC Non-response

**Figure 2:**
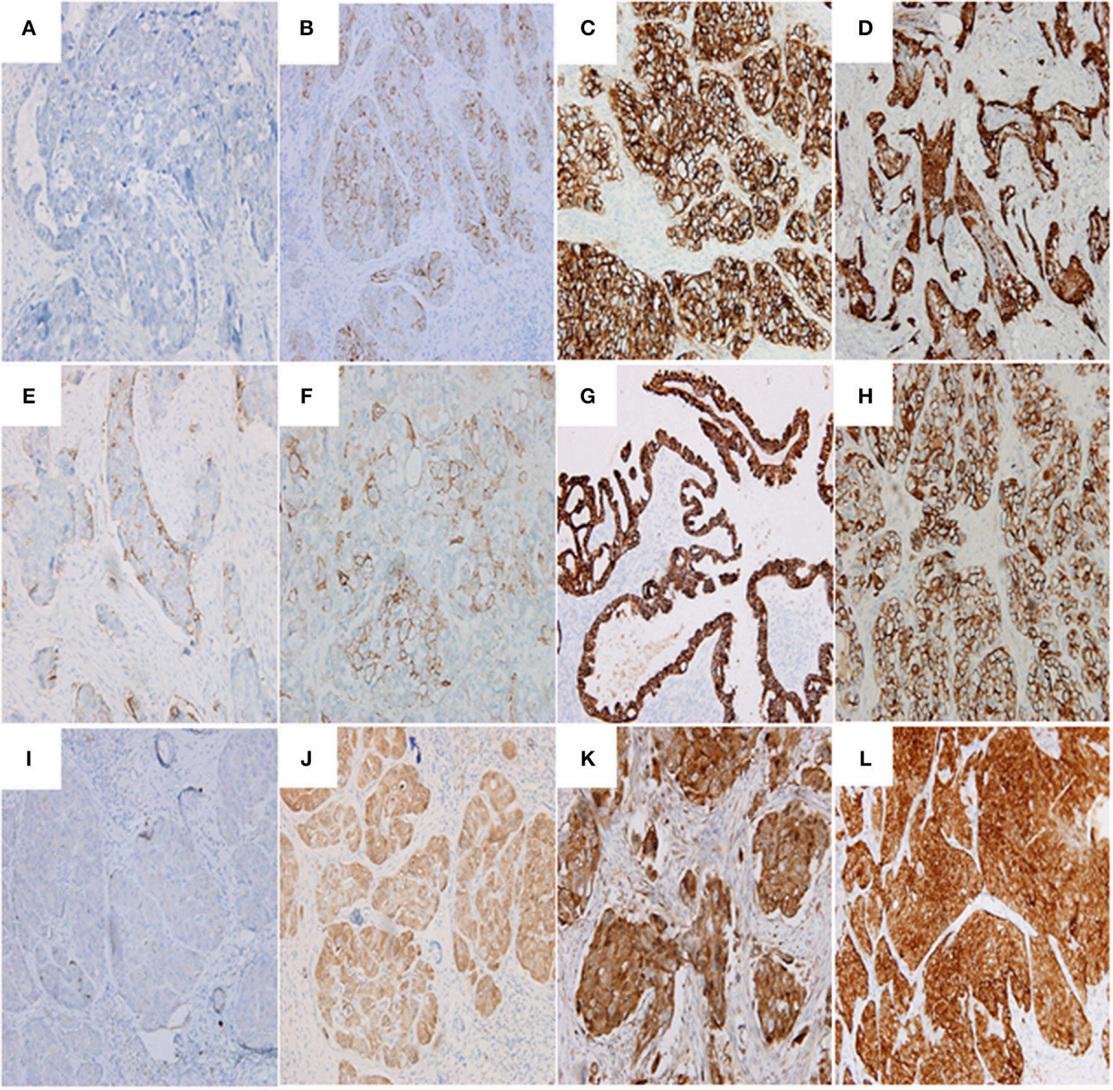
Representative images showing PD-L1 **(A–D)**, SEMA4D **(E–H)**, and VEGF **(I–L)** expressions. **(A)** PD-L1 negative expression in serous ovarian adenocarcinoma (G3, FIGO Stage II) (200 ×); **(B)** serous ovarian adenocarcinoma (G3, FIGO Stage III) with immunostaining of PD-L1 in the membrane of cancer cells (200 ×; Evaluation: 3 points); **(C)** serous ovarian adenocarcinoma (G3, FIGO Stage III) with immunostaining of PD-L1 in the membrane and cytoplasm of cancer cells (200 ×; Evaluation: 5 points); **(D)** serous ovarian adenocarcinoma (G3, FIGO Stage IV) with immunostaining of PD-L1 (200 ×; Evaluation: 5 points); **(E)** serous ovarian adenocarcinoma (G2, FIGO Stage III) with immunostaining of SEMA4D (200 ×; Evaluation: 2 points); **(F)** ovarian clear cell carcinoma (G3, FIGO Stage III) with immunostaining of SEMA4D (200 ×; Evaluation: 3 points); **(G)** endometrioid ovarian adenocarcinoma (G2, FIGO Stage II) with immunostaining of SEMA4D in the membrane of cancer cells (200 ×; Evaluation: 5 points); **(H)** serous ovarian adenocarcinoma (G3, FIGO Stage III) with immunostaining of SEMA4D in the membrane of cancer cells (200 ×; Evaluation: 5 points); **(I)** serous ovarian adenocarcinoma (G3, FIGO Stage II) with negative immunostaining of VEGF (200 × ); **(J)** serous ovarian adenocarcinoma (G3, FIGO Stage II) with immunostaining of VEGF in the cytoplasm of cancer cells (200 ×; Evaluation: 3 point); **(K)** serous ovarian adenocarcinoma (G3, FIGO Stage III) with immunostaining of VEGF (200 ×; Evaluation: 5 points); **(L)** serous ovarian adenocarcinoma (G3, FIGO Stage III) with immunostaining of VEGF (400 ×; Evaluation: 7 points).

Immunohistochemistry revealed that 71.43% (45/63), 50.79% (32/63), and 58.73% (37/63) of ovarian cancer tissues with BC response stained positively for SEMA4D, VEGF and PD-L1, which were significantly higher than the positive staining in the group of ovarian cancer tissues with BC non-response (71.43% vs. 49.18%, 50.79% vs. 31.15%, and 58.73% vs. 39.34%, *p* < 0.05, respectively; Table 2). Figure 2 shows the representative immunohistochemistry results.

**Table 2:** PD-L1, SEMA4D, and VEGF expressions in ovarian cancer tissues.

**Group**	**Cases (N)**	**SEMA4D positive expression *N (%)***	***P***	**VEGF positive expression *N (%)***	***P***	**PD-L1 positive expression *N (%)***	***P***
EOC tissues with BC response	63	45 (71.43)	0.017	32 (50.79)	0.030	37 (58.73)	0.033
EOC tissues with BC non-response	61	30 (49.18)		19 (31.15)		24 (39.34)	

*EOC, epithelial ovarian cancer*.

*BC, bevacizumab plus single regimen chemotherapy*.

### Positive Correlation Between the Expression of PD-L1 and SEMA4D or VEGF in Ovarian Cancer With BC Response

Among 75 EOC tissues stained positively for SEMA4D, 50.67% (38/75) stained positively for VEGF and 64.00% (48/75) stained positively for PD-L1. Likewise, among 61 EOC tissues stained positively for PD-L1, 60.66% (37/61) stained positively for VEGF. The correlation of SEMA4D, VEGF and PD-L1 expressions were identified to be closely by Spearman correlation (*r* = 0.233, 0.344 and 0.363, *P* < 0.05, respectively, see Table 3) and Mantel-Haenszel test (χ^2^ = 6.119, 15.060, and 17.213, *P* < 0.05, respectively, see Table 2).

**Table 3:** Relationship of PD-L1, VEGF, and SEMA4D expressions in EOC tissues.

		**Cases (N)**	**VEGF expression N (%)**		**Spearman correlation**		**Mantel-Haenszel test**		**PD-L1 expression N (%)**		**Spearman correlation**		**Mantel-Haenszel test**	
					***r***	***P***	**χ^2^ Value**	***P***			***r***	***P***	**χ^2^ Value**	***P***
			**Negative**	**Positive**	**0.233**	**0.008**	**6.119**	**0.013**	**Negative**	**Positive**	**0.344**	**<0.001**	**15.060**	**<0.001**
SEMA4D expression N (%)	Negative	49	36 (73.47)	13 (26.53)					36 (73.47)	13 (26.53)				
	Positive	75	37 (49.33)	38 (50.67)					27 (36.00)	48 (64.00)				
					0.363	<0.001	17.213	<0.001						
PD-L1 expression N (%)	Negative	63	49 (77.78)	14 (22.22)										
	Positive	61	24 (39.34)	37 (60.66)										

### Expression of PD-L1, VEGF, and SEMA4D and Clinicopathologic Characteristics of EOC Patients

As shown in Table 4, over-expressions of SEMA4D, VEGF, and PD-L1 were closely related to EOC tissues with lymph node (LN) metastasis and patients' response to BC (*P* < 0.05). Furthermore, over-expression of SEMA4D was also related to low histologic grade, residual disease ≥1 cm, and CA125 > 573.35 U/ml (all of them *P* < 0.05). Over-expression of VEGF was closely related to EOC tissues with advanced FIGO stage and ascites volume >2,000 mL (both of them *P* < 0.05). Over-expression of PD-L1 was closely related to EOC tissues with low histologic grade, advanced FIGO stage and ascites volume >2,000 mL (all of them *P* < 0.05).These results suggest that over-expression of PD-L1, VEGF and SEMA4D are associated with more malignant EOC phenotypes.

**Table 4:** Correlation between PD-L1, VEGF, and SEMA4D expressions with clinicopathologic characteristics of EOC patients.

**Variable**	**Cases (N)**	**SEMA4D expression (N)**		***P***	**VEGF expression (N)**		***P***	**PD-L1 expression (N)**		***P***
		**Negative**	**Positive**		**Negative**	**Positive**		**Negative**	**Positive**	
Age				0.463			0.466			0.150
≤52 years	62	27	35		39	23		36	26	
>52 years	62	22	40		34	28		27	35	
Menopausal status				0.457			0.852			0.362
Yes	76	28	48		44	32		36	40	
No	48	21	27		29	19		27	21	
Pathologic type				0.848			0.848			0.347
Serous carcinoma	82	33	49		49	33		39	43	
Mucous and others	42	16	26		24	18		24	18	
Histologic grade				0.001			0.060			0.027
G_1_−_2_	47	28	19		33	14		30	17	
G_3_ or undifferentiated	77	21	56		40	37		33	44	
FIGO Stage				0.093			<0.001			0.001
I–II	51	25	26		43	8		35	16	
III–IV	73	24	49		30	43		28	45	
LN metastasis				0.014			0.025			<0.001
No	76	37	39		51	25		50	26	
Yes	48	12	36		22	26		13	35	
Residual disease				0.010			0.835			0.148
<1 cm	93	43	50		54	39		51	42	
≥1 cm	31	6	25		19	12		12	19	
Ascites volume (mL)				0.628			0.007			0.016
<2,000	103	42	61		55	48		47	56	
≥2,000	21	7	14		18	3		16	5	
Serum CA125 (U/mL)				0.040			0.199			0.103
<573.35	74	35	39		40	34		33	41	
≥573.35	50	14	36		33	17		30	20	
Patients' response to BC				0.017			0.030			0.033
Response (CR, PR and SD)	63	18	45		31	19		26	37	
Non-response (PD)	61	31	30		42	32		37	24	

*BC, bevacizumab plus single regimen chemotherapy; CR, Complete Response; PR, Partial Response; PD, Progressive Disease; SD, Stable Disease*.

### Survival Analysis of Prognosis Factors in EOC

**Figure 3:**
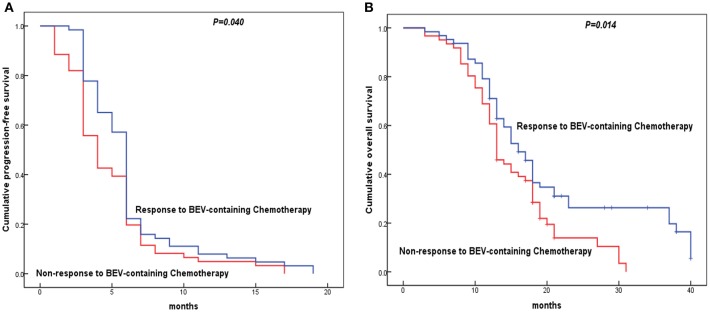
Kaplan-Meier analysis for the disease-free survival and overall survival of epithelial ovarian cancer (EOC) patients with cisplatin resistant recurrence according to patients' response to Bevacizumab-containing chemotherapy. The survival curves were analyzed by the log rank test. **(A)** Cisplatin resistant EOC patients with non-response to Bevacizumab-containing chemotherapy showed shorter progress-free survival than those with response to Bevacizumab; **(B)** Cisplatin resistant EOC patients with non-response to Bevacizumab-containing chemotherapy showed shorter overall survival than those with response to Bevacizumab.

The results indicated the shorter median PFS and OS were associated with low histologic grade, advanced stage, lymph node metastasis, residual disease ≥1 cm, VEGF positive expression, SEMA4D positive expression, PD-L1 positive expression, and patients' response to BC (all of them *P* < 0.05, Table 5). Moreover, EOC patients with menopause status had a shorter median PFS (*P* = 0.042, Table 5). Variables that were significant in the univariate analysis were examined by multivariate analysis. We found the patients' response to BC was the independent factor for evaluation of PFS and OS in the Cox proportional hazard model (*P* < 0.05, Table 5, Figure 3). Additionally, LN metastasis was an independent factor for evaluation of PFS, as well as FIGO stage and SEMA4D expression were independent factors for evaluation of OS in this study.

**Table 5:** Univariate and multivariate survival analyses of the prognostic factors for overall and disease-free survival in EOC patients.

**Variable**	**Cases (N)**	**Progression-free survival**					**Overall survival**				
		**Univariate analysis**		**Multivariate analysis**			**Univariate analysis**		**Multivariate analysis**		
		**Media of PFS**	***P^***a***^***	**HR**	**95% CI for HR**	***P^***b***^***	**Media of OS**	***P^***a***^***	**HR**	**95% CI for HR**	***P^***b***^***
Age (years)			0.532	NA	NA	NA		0.147	NA	NA	NA
≤52	62	6					17				
>52	62	4					13				
Menopause			0.042	0.686	0.467–1.006	0.054		0.725	NA	NA	NA
Yes	76	4					15				
No	48	6					13				
Pathologic type			0.088	NA	NA	NA		0.777	NA	NA	NA
Serous	82	4					15				
Mucous and others	42	6					15				
Histologic grade			0.012	1.015	0.651–1.583	0.947		0.017	1.208	0.716–2.037	0.479
G_1−2_	47	6					17				
G_3_	77	5					13				
FIGO Stage			0.001	0.829	0.487–1.409	0.488		<0.001	0.560	0.319–0.984	0.044
I–II	51	6					20				
III–IV	73	4					13				
LN metastasis			<0.001	0.523	0.328–0.836	0.007		<0.001	0.645	0.385–1.081	0.096
No	76	6					18				
Yes	48	3					12				
Residual tumor			0.041	1.349	0.862–2.112	0.190		0.001	1.447	0.875–2.393	0.150
<1 cm	93	6					18				
≥1 cm	31	4					12				
Ascites volume (ml)			0.576	NA	NA	NA		0.608	NA	NA	NA
≤2,000	103						15				
>2,000	21						14				
Serum CA125 (U/ml)			0.374	NA	NA	NA		0.070	NA	NA	NA
≤573.35	74	6					17				
>573.35	50	5					13				
VEGF expression			0.049	1.396	0.864–2.255	0.172		0.036	1.208	0.733–1.990	0.458
Negative	73	6					17				
Positive	51	5					13				
SEMA4D expression			0.003	1.448	0.937–2.238	0.096		<0.001	2.561	1.493–4.392	0.001
Negative	49	6					19				
Positive	75	5					13				
PD-L1 expression			0.025	0.834	0.517–1.344	0.455		0.047	0.942	0.579–1.534	0.811
Negative	63	6					18				
Positive	61						13				
Patients' response to BC		4	0.040	0.528	0.350–0.799	0.002		0.014	0.409	0.263–0.636	<0.001
Response (CR, PR and SD)	63	6					16				
Non-response (PD)	61	4					13				

*P^a^, P value, log rank test; HR, hazard ratio; CI, confidence interval; P^b^, P value, Cox regression; CR, complete response; PR, partial response; SD, stable disease; PD, progressive disease; NA, not applicable; BC, bevacizumab plus single regimen chemotherapy*.

### Atezolizumab in Combination With Bevacizumab Inhibit Proliferation, Migration, and Invasion of Cisplatin Resistant Ovarian Cancer Cell Line A2780cis Synergistically

**Figure 4:**
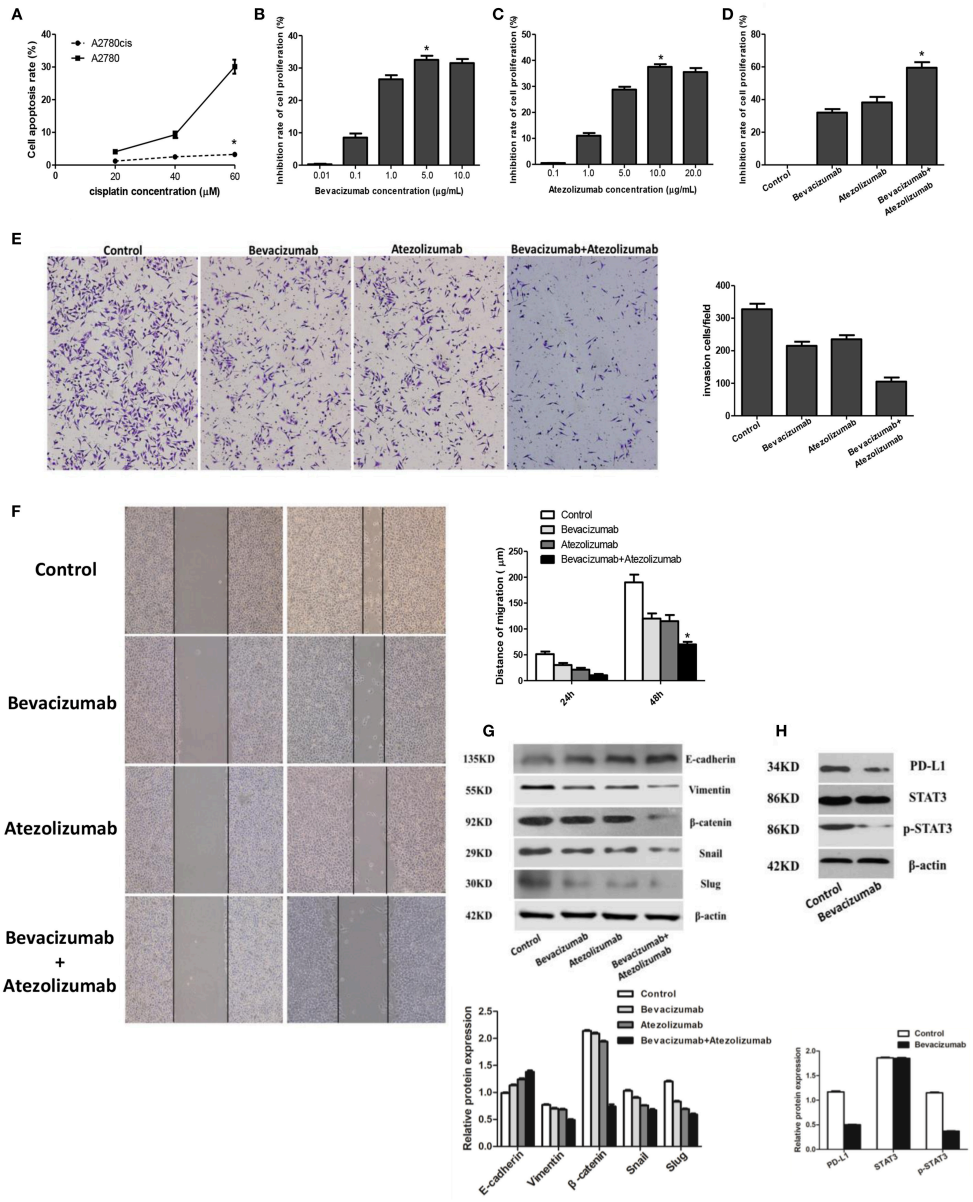
Atezolizumab in combination with Bevacizumab inhibit proliferation, migration, and invasion of cisplatin resistant ovarian cancer cell line A2780cis synergistically, which may be related to Bevacizumab suppresses the epithelial-mesenchymal transition (EMT) and PD-L1 expression by targeting STAT3. **(A)** Cell apoptosis rate of A2780cis was significantly lower than A2780 (^*^P < 0.05), which proved A2780cis was the cisplatin-resistant cell line. **(B)** Based the different concentrations (0.01, 0.1, 1.0, 5.0, and 10.0μg/mL) of Bevacizumab to treat A2780cis, the proliferation of cells was obviously inhibited by using of 5.0 μg/mL Bevacizumab (^*^P < 0.05). **(C)** Based the different concentrations (0.1, 1.0, 5.0, 10.0, and 20.0 μg/mL) of Atezolizumab to treat A2780cis, the proliferation of cells was obviously inhibited by using of 10.0 μg/mL Atezolizumab (^*^P < 0.05). **(D)** The inhibition rate of A2780cis cell proliferation was dramatically increased after treated by Bevacizumab (5.0 μg/mL) and Atezolizumab (10.0 μg/mL) using CCK-8 assay (^*^P < 0.05). The wound healing assays **(E)** and transwell assay **(F)** showed that Bevacizumab and Atezolizumab impaired A2780cis cell migration and invasion, respectively (^*^P < 0.05). **(G)** Mesenchymal marker proteins (vimentin and β-catenin) and EMT-mediating transcription factor (Snail and Slug) were down-regulated in A2780cis cells after treated by Bevacizumab or/and Atezolizumab, while the epithelial marker E-cadherin was up-regulated, especially under the combination of them. **(H)** The activation of STAT3 and expression of PD-L1 in A2780cis cells were decreased by Bevacizumab treatment. Thus, these findings strongly suggested that Bevacizumab inhibit EMT and PD-L1expression via down-regulating the phosphorylation of STAT3.

After treated with different concentration of cisplatin (20, 40, and 60 μmol /L) 24 h, the cell apoptosis rate of A2780cis was 1.22 ± 0.07, 2.50 ± 0.15, and 3.25 ± 0.35 respectively using flow cytometry detection, compared with the cell apoptosis rate of A2780 was 4.10 ± 0.43, 9.27 ± 0.99, and 30.12 ± 2.14. Thus, the cell apoptosis rate of A2780cis was significantly lower than A2780 (*P* < 0.05), which proved A2780cis was the cisplatin-resistant cell line (Figure 4A). And then, BEV with different concentrations (0.01, 0.1, 1.0, 5.0, and 10.0 μg/mL) were used to treat A2780cis to know the proliferation of cells under anti-angiogenesis treatment. The results identified the proliferation of cells was obviously inhibited by using of 5.0 μg/mL Bevacizumab treating A2780cis, which indicated this concentration of Bevacizumab was used to perform the following experiments (*P* < 0.05, Figure 4B). Similarly, the concentration of Atezolizumab 10.0 μg/mL was selected as the maximum for further investigations (Figure 4C). Furthermore, the inhibition rate of A2780cis cell proliferation was dramatically decreased after treated by Bevacizumab (5.0 μg/mL) and Atezolizumab (10.0 μg/mL) using CCK-8 assay (59.50 ± 3.42%, *P* < 0.05, Figure 4D). Additionally, wound healing assays and transwell assay showed that Bevacizumab and Atezolizumab impaired A2780cis cell migration and invasion, respectively (Figures 4E,F).

### Bevacizumab Suppresses the Epithelial-Mesenchymal Transition and Pd-L1 Expression in Cisplatin Resistant Ovarian Cancer Cell A2780cis by Targeting STAT3

Furthermore, it reached a consensus that the EMT is a pivotal procedure for epithelial cells to acquire mesenchymal phenotype to promote tumor metastasis. we detected epithelial-mesenchymal transition (EMT) markers and found that mesenchymal marker proteins (vimentin and β-catenin) and associated EMT transcription factors (Snail and Slug) were downregulated in A2780cis cells after treated by Bevacizumab or/and Atezolizumab, while the epithelial marker E-cadherin was up-regulated, especially under the combination of them (Figure 4G), which indicated Bevacizumab and Atezolizumab inhibit EMT in cisplatin resistant ovarian cancer cells. Additionally, former data had already illustrated that signal transducer and activator of transcription 3 (STAT3) was a significant inducer of EMT by the transcriptional activation of slug and could also regulate PD-L1expression. Our results analogously demonstrated the activation of STAT3 and expression of PD-L1 in A2780cis cells were decreased by Bevacizumab treatment (Figure 4H). Thus, these findings strongly suggested that Bevacizumab inhibit EMT and PD-L1expression *v*ia down-regulating the phosphorylation of STAT3.

### Bevacizumab and Atezolizumab Induced Synergistic Anti-tumor Effect *in vivo*

**Figure 5:**
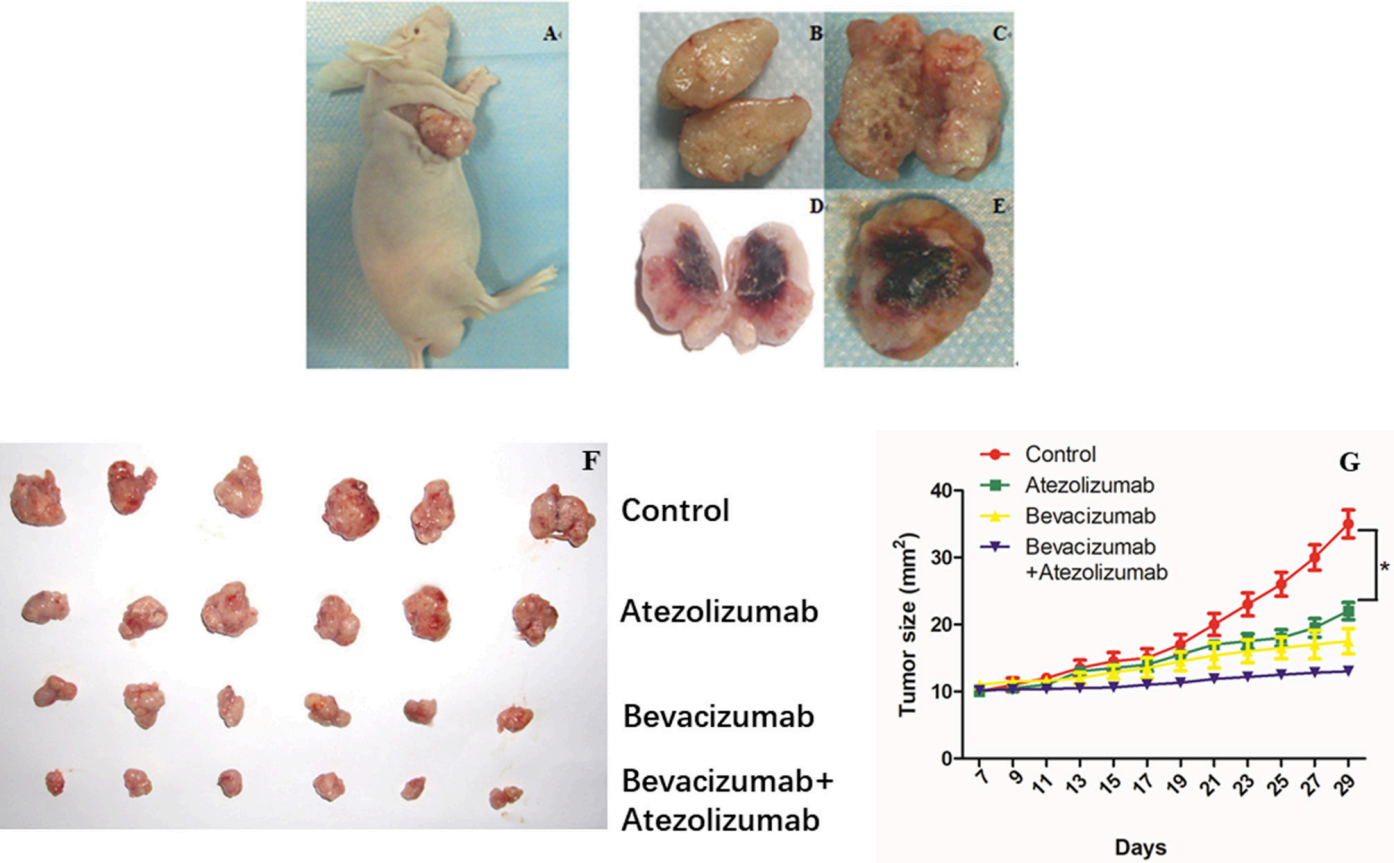
Simultaneous Bevacizmab combining Atezolizumab therapeutic strategy induced synergistic anti-tumor effect *in vivo*. **(A)** Female BALB/C nude mice were inoculated subcutaneously with A2780cis cells and were given with IgG as control, Bevacizumab (5 mg/kg) every 48 h, Atezolizumab (10 mg/kg) every 48 h, Bevacizumab (5 mg/kg) + Atezolizumab (10 mg/kg) every 48 h. **(B–E)** Tumors were cut open longitudinally after they were taken out from killed mice. **(F)** Isolated tumors from four different groups were showed neatly. **(G)** Data are presented as mean ± standard deviation of 6 mice of each group. ^*^P < 0.05.

We investigated the efficacy of simultaneous blockade with both VEGF and PD-L1 *in vivo* using the murine model. *In-vivo* treatment either with Bevacizumab or Atezolizumab induced a substantial anti-tumor effect and inhibited tumor growth significantly compared to control (*P* < 0.05, Figure 5). There was no significant difference in tumor growth between Bevacizumab and Atezolizumab treatment (*P* = 0.1256, Figure 5). Furthermore, dual blockade by Bevacizumab and Atezolizumab inhibited tumor growth significantly compared to each treatment (*P* < 0.05, Figure 5). Thus, the combination therapy of anti-PD-L1 and anti-VEGF showed a synergistic anti-tumor effect in tumor growth.

## Discussion

Angiogenesis is a complex process controlled by certain biomolecules produced in the body (12). Physiological angiogenesis processes are crucial during embryo development, wound healing, and collateral formation for improved organ perfusion (13). However, abnormally accelerated angiogenesis processes or pathological angiogenesis are associated with various disorders and diseases, especially for carcinogenesis. Tumor mess cannot exceed 2mm without neovascularization to provide oxygen and nutrients. VEGF, a powerful angiogenic agent in neoplastic tissues, has been widely studied in the field of neoplastic vascularization (14). Previous researches had confirmed the expression of VEGF was increased in many cancers, including colon cancer (15), lung cancer (16), breast cancer (17), and gastric cancer (18), which indicated the poor survival for patients. Bevacizumab, a recombinant humanized monoclonal antibody to VEGF and known by its brand name, Avastin®, blocks tumor cell-derived VEGF-A, impairing the development of new vessels and leading to tumor starvation and, consequently, growth inhibition (19). Preclinical studies have shown that Bevacizumab can potentially enhance the delivery of chemotherapeutic agents by normalizing blood vessels and reducing interstitial fluid pressure (20, 21). The well-known AURELIA trial compared the efficacy of single-regimen chemotherapy vs. chemotherapy combined with Bevacizumab in platinum-resistant ovarian cancer. The results found that the combination therapy including Bevacizumab improved PFS over chemotherapy alone (22).

The 124 EOC patients, diagnosed with cisplatin resistance due to progressing <6 months after completing platinum-based therapy, were retrospectively enrolled in this study. Based on responding to at least 2 BC cycles, these Patients were divided into BC response group and BC non-response group. The expressions of VEGF and SEMA4D, two crucial proteins promoting tumor angiogenesis, were detected in ovarian cancer tissues by immunohistochemistry. We found that the positive expressions of VEGF and SEM4D in ovarian cancer tissues with BC response group were both significantly higher than that in the group of ovarian cancer tissues with BC non-response. Over-expressions of SEMA4D and VEGF were closely related to EOC tissues with LN metastasis and patients' response to BC, which showed a poor prognosis for ovarian cancer patients.

As for SEMA4D, accumulating evidences illustrated it was a potent inducer of angiogenesis, and its overexpression was associated with tumor progression and poor prognosis in a variety of malignancies (9, 23, 24). A series of studies accomplished by Zhou and the colleagues explained the SEMA4D in combination with VEGF to promote cancer progression synergistically. They deemed anti-VEGF strategies can lead to upregulation of other pro-angiogenic factors that can compensate for the loss of VEGF, eventually leading to failure of therapy (25). They demonstrated that expression of SEMA4D, which was under the control of the HIF-family of transcription factors, cooperated with VEGF to promote tumor growth and vascularity in oral squamous cell carcinoma (OSCC) (26). They courageously suggested that targeting SEMA4D function along with VEGF could represent a novel anti-angiogenic therapeutic strategy for the treatment of solid tumors (27). Our results also showed the correlation of SEMA4D and VEGF positive expressions were closely in EOC tissues, which hinted targeting SEMA4D might serve as a parallel option for traditional antiangiogenic therapy with single regimen in ovarian cancer.

Cancer immunotherapy has made great strides in the recent decade, especially in the area of immune checkpoint blockade (28). The development of immune checkpoint inhibitor represented by PD-L1 has provided new hope for the treatment of advanced cancer patients (29). PD-L1, also known as B7-H1 or CD274, is encoded by the PDCDL1 gene on human chromosome 9 and also the first functionally characterized ligand for PD-1, which plays a key role in maintaining peripheral and central immune cell tolerance by binding to PD-1 receptors (30). Many researchers have found that the expression level of PD-L1 might be an effective predictive biomarker for immunotherapy, especially in the treatment using PD-L1 inhibitors (31, 32). In this study, we found the positive expression of PD-L1 in ovarian cancer tissues with BC response is obviously higher than the positive staining in the group of ovarian cancer tissues with BC non-response. Over-expression of PD-L1 was closely related to EOC tissues with low histologic grade, advanced FIGO stage and ascites volume >2,000 mL, LN metastasis and patients' response to BC. PD-L1 positive expression indicated the shorter median PFS and OS for ovarian cancer patients. Our results were similar with previous studies, which have reported data regarding the association between PD-L1 status and prognosis in cancer, indicating a worse outcome and late stage for patients with high PD-L1 expression (33–37).

At present, immunotherapy in combination with anti-angiogenesis therapy were expected to be a promising synergetic strategy in cancer treatment (38). Broadly speaking, preclinical studies suggest that tumor-associated blood and lymphatic vascular formation promote an immunosuppressive microenvironment by modulating the recruitment, adhesion, trafficking, and function of immune cells in several ways (39). In preclinical models, inhibition of VEGF signaling promoted antitumor immunity and may enhance the efficacy of immune checkpoint blockade (40). In renal cell carcinoma (RCC), the combination of Bevacizumab plus Atezolizumab has advanced rapidly through clinical development. A phase IB trial of bevacizumab plus Atezolizumab in 12 patients with previously untreated metastatic RCC reported the promising objective response ratio (ORR) reached 40% in the first 10 patients evaluated (40). Excitingly, our results found the correlation of SEMA4D, VEGF, and PD-L1 expression were closely in CREOC tissues. Consequently, cell functional assays showed that Bevacizumab and Atezolizumab impaired the proliferation, migration and invasion of cisplatin ovarian cancer cell line A2780cis *in vitro* synergistically. Moreover, the combination therapy of anti-PD-L1 and anti-VEGF showed a synergistic anti-tumor effect of tumor growth *in vivo*. These results provided the basis to perform the Bevacizumab plus Atezolizumab therapeutics for advanced CREOC patients.

EMT has been proven to be an important early event of tumor cell metastatic dissemination, in which cells are endowed with a more motile and invasive potential (41, 42). Previously, Huang W et al. reported Bevacizumab induced EMT phenotype in glioblastoma cell line U87MG and the overexpression of BATF2 (basic leucine zipper ATF-like transcription factor 2), a multi-target transcriptional repressor, significantly inhibited Bevacizumab-induced EMT with suppression of Wnt/β-catenin signaling (43). In pancreatic cancer, Carbone et al. established and validated two murine models of human pancreatic cancer resistant to Bevacizumab *in vivo*. They found that secreted factors overexpressed by Bevacizumab-resistant pancreatic cancer cells acted in an autocrine manner to induce EMT and were responsible for increased aggressiveness of Bevacizumab-resistant pancreatic tumors. Thus, they suggested EMT markers as potential biomarkers for selecting patients with pancreatic cancer for antiangiogenic therapy (44). As for Atezolizumab, Thar Min AK et al. showed that PD-L1 expression on tumor cells was positively correlated with EMT status in esophageal squamous cell carcinoma (ESCC) and EMT-converted ESCC indicated the upregulation of PD-L1 at both protein (total and surface) and mRNA levels, which provided a strong rationale for the clinical use of Atezolizumab for ESCC patients (45). Similarly, Funaki and the colleagues illustrated PD-L1 is correlated with EMT status. PD-L1 expression after induction therapy was significantly higher compared to before induction therapy and was correlated with the EMT change. PD-L1 may be upregulated during EMT, and anti-PD-L1 immunotherapy might provide reliable treatment of thymic carcinoma in combination with chemotherapy (46).

In our study, mesenchymal marker proteins (vimentin and β-catenin) and associated EMT transcription factors (Snail and Slug) were downregulated in A2780cis cells after treated by Bevacizumab or/and Atezolizumab, while the epithelial marker E-cadherin was up-regulated, especially under the combination of them. STAT3 was identified to be a significant inducer of EMT by the transcriptional activation of slug and could also regulate PD-L1expression in gastric cancer (47), which was analogously demonstrated to our results in A2780cis cells. Our findings strongly evidenced this hypothesis that that combining checkpoint inhibitor immunotherapies with antiangiogenic treatment may have a synergistic effect and enhance the efficacy of both treatments.

However, there are limitations of our study. Firstly, our study just is a retrospective summarization of the patients' literature of our center and lack rich experiences of the application of Atezolizumab, there is a great need for prospective and large-scale studies to evaluate the safety and efficacy of Bevacizumab in combination with Atezolizumab as a feasible strategy for advanced recurrent EOC patients with cisplatin resistance. Secondly, the underlying mechanism between anti-angiogenesis and immunotherapy remain largely uninvestigated. More detailed mechanical explorations *in vivo* warrant to perform.

Conclusively, all of the expressions of PD-L1, VEGF, and SEMA4D in CREOC tissues with BC response were found significantly higher than that in the BC non-response group and PD-L1 expression correlated with SEMA4D and VEGF positively. Over-expressions of PD-L1, VEGF and SEMA4D are associated with more malignant clinicopathologic characteristics of CREOC Patients. The response to BC was the independent factor for evaluation of PFS and OS for recurrent ovarian cancer patients with cisplatin resistance. Atezolizumab in combination with Bevacizumab inhibited the proliferation, migration, and invasion of cisplatin resistant ovarian cancer cell *in vitro* synergistically via Bevacizumab suppressing EMT and PD-L1 expression by targeting STAT3. Moreover, the combination therapy of Bevacizumab and Atezolizumab induced synergistic anti-tumor effect *in vivo*. Our findings suggest a novel therapeutic strategy for advanced recurrent EOC patients with cisplatin resistance and its mechanism is deserved to study further in the future.

## Author Contributions

YC designed this study. LZ completed statistics and wrote this paper. FL completed cell functional assays. LB and WL collected the data and completed related assay.

### Conflict of Interest Statement

The authors declare that the research was conducted in the absence of any commercial or financial relationships that could be construed as a potential conflict of interest.
